# Impact of Working Memory Load on Cognitive Control in Trait Anxiety: An ERP Study

**DOI:** 10.1371/journal.pone.0111791

**Published:** 2014-11-04

**Authors:** Senqing Qi, Qinghong Zeng, Yangmei Luo, Haijun Duan, Cody Ding, Weiping Hu, Hong Li

**Affiliations:** 1 MOE Key Laboratory of Modern Teaching Technology, Shaanxi Normal University, Xi'an, China; 2 Department of Psychology, The Chinese University of Hong Kong, Shatin, N.T., Hong Kong; 3 School of Psychology, Shaanxi Normal University, Xi'an, China; 4 Institute of Safety Psychology, Liaoning University of Engineering and Technology, Fuxin, China; 5 Research Centre for Brain Function and Psychological Science, Shenzhen University, Shenzhen, China; Utrecht University, Netherlands

## Abstract

Whether trait anxiety is associated with a general impairment of cognitive control is a matter of debate. This study investigated whether and how experimentally manipulated working memory (WM) load modulates the relation between trait anxiety and cognitive control. This question was investigated using a dual-task design in combination with event-related potentials. Participants were required to remember either one (low WM load) or six letters (high WM load) while performing a flanker task. Our results showed that a high WM load disrupted participants' ability to overcome distractor interference and this effect was exacerbated for the high trait-anxious (HTA) group. This exacerbation was reflected by larger interference effects (i.e., incongruent minus congruent) on reaction times (RTs) and N2 amplitudes for the HTA group than for the low trait-anxious group under high WM load. The two groups, however, did not differ in their ability to inhibit task-irrelevant distractors under low WM load, as indicated by both RTs and N2 amplitudes. These findings underscore the significance of WM-related cognitive demand in contributing to the presence (or absence) of a general cognitive control deficit in trait anxiety. Furthermore, our findings show that when limited WM resources are depleted by high WM load, HTA individuals exhibit less efficient recruitments of cognitive control required for the inhibition of distractors, therefore resulting in a greater degree of response conflict.

## Introduction

Trait anxiety, which is a vulnerable personality factor for anxiety disorders and depression [1], [2], is defined as the disposition to experience frequent and intense anxiety and worry in response to various stress situations [3]. Individuals with high-trait-anxiety (HTA) often suffer from excessive, uncontrollable, and irrational worry about uncertain events, as well as difficulty in focusing their attention on the tasks at hand. Certain symptoms may be mediated by impaired cognitive control of task-irrelevant distracters [4], [5]. Therefore, investigating the influence of trait anxiety on cognitive control may provide insights into the nature of trait anxiety and influence the effective prevention of anxiety disorders.

Although many studies suggest that trait anxiety is associated with impaired cognitive control of threat distractors (for a review, see [6]), recent studies have been demonstrating that this impairment can even be observed in the absence of threat (e.g., [4], [7]–[10]). Recent findings may be accounted for within the framework of attentional control theory (ACT) [5], [11], which posits that trait anxiety is associated with a general impairment of cognitive control. However, the data showed that such impairment sometimes only manifested in neural processes and not in behavioral performance (e.g., [12]–[14]), and sometimes even disappeared completely (e.g., [15]–[17]). Therefore, whether trait anxiety is associated with a general impairment of cognitive control is a matter of debate.

The modified ACT further proposes that task demands on cognitive resources are primary determinants of whether impaired cognitive control will be observed for HTA individuals [11]. Specifically, when tasks place relatively high demands on cognitive resources, HTA individuals are expected to show impaired cognitive control. Under a moderate level of task demands on cognitive resources, HTA individuals may show impaired processing efficiency but not disrupted cognitive control on behavioral performance. In such condition, HTA individuals compensate for their impaired processing efficiency by making more efforts and recruiting greater attentional resources, therefore achieving comparable task performance with low-trait-anxiety (LTA) individuals. The level of perceptual load has been shown to determine whether HTA individuals show impaired inhibition of task-irrelevant distractors, although the conclusions are inconsistent [4], [18]. For instance, Sadeh and Bredemeier [18] have shown that anxiety is related to difficulty in inhibiting irrelevant distractors under high, but not low, perceptual load. In addition to the level of perceptual load, the load theory of attention [19] advocates that placing high WM load on tasks seriously impacts the efficiency of cognitive control. For example, the flanker interference effect was greater under high WM than under low WM load (e.g., [20]–[22]). The flanker interference effect refers to the longer reaction times (RTs) or more errors in incongruent trials than in congruent trials. Studies therefore suggested that sufficient WM resources are essential for overcoming distractor interference and for optimal selective attention performance.

A previous WM model posits that WM is a limited-capacity system for active representations of a set of objects, and that its information storage function is associated with the specialized neural activity of the prefrontal cortex (PFC) during WM delay-period [23]. However, a recent model proposes that WM functions arise through the coordinated recruitment, by attention, of neural systems responsible for the representation of sensory and action information. Hence, the activity of PFC during WM delay-period is not associated with the temporary storage of information, but is associated with the general executive control processes, including the flexible mediation of internal or external interference [24]–[26]. At the neural level, a high degree of overlap exists between the regions activated by WM storage and cognitive control, including dorsolateral PFC, anterior cingulate, and parietal cortex [27], [28]. Specifically, studies that examine WM have found that the activation of the dorsolateral PFC is greater under high WM than under low WM load [20], [29]. In response interference tasks, both dorsolateral PFC and dorsal anterior cingulate cortex (ACC) play important roles in detecting conflict and implementing cognitive control, although the time course of relevant activity in these brain regions remains debatable [30], [31]. Therefore, recent models and evidence show that WM is similar to flexibly deployable attention and is indistinguishable from cognitive control.

Related to the present study, trait anxiety interferes with the recruitment of the DLPFC in tasks involving inhibiting task-irrelevant distracters [4], [11], [12]. Therefore, WM-related cognitive demand and cognitive control of distractors seem to compete for the recruitment of the dorsolateral PFC in HTA individuals. If the previously greater activation of the dorsolateral PFC under high WM load leads to subsequently reduced recruitment of the dorsolateral PFC required for inhibition control of task-irrelevant information, the flanker interference effects under high WM load should be greater for HTA individuals than for LTA individuals. However, few of the aforementioned studies tested the impact of WM load on cognitive control of emotionally neutral distractors in trait anxiety, with the exception of the behavioral study of Berggren and colleagues, which demonstrated that anxiety was associated with the magnitude of cost (i.e., anti-saccade minus pro-saccade latencies) on inhibition under high WM load in an anti-saccade task [32]. However, doubt remains as to whether the level of WM load affected memory task performance differently in the HTA and LTA groups because the accuracy of the WM task performance was not recorded in the aforementioned study.

The present study was designed to further test whether and how experimentally manipulated WM load modulates the relation between trait anxiety and cognitive control at the behavioral and neural levels. First, this problem is an important issue to explore because it elucidates the contextual factors that moderate anxiety-related performance deficits. Second, whether HTA individuals can efficiently deal with tasks that carry a strong cognitive burden by ignoring distracting stimuli is highly relevant to their daily life. Therefore, the answer to this issue might provide useful guidance for HTA individuals in addressing their daily problems.

We used a dual-task design to control the amount of information stored in WM (one or six letters) while participants performed a flanker interference task. For the flanker task, the conflict-related N2 of the scalp-recorded event-related potential (ERP) is reflective of fronto-limbic mediated conflict processing in cognitive control [33]–[36]. Compared with congruent trials, incongruent trials elicit a more negative (i.e., larger) N2 at fronto-central regions, appearing from approximately 250 to 350 ms post-stimulus. The amplitude of N2 is associated with conflict strength resulting from competition between task-relevant and task-irrelevant inputs [34], [37]. Furthermore, the neural signal indexed by N2 is sensitive to the adjustment of cognitive control because the recruitment of increased cognitive resources leads to N2 reduction on the succeeding trials [33], [36]. The neural generator of the N2 likely lies in the medial PFC, and more specifically, in the ACC [38]. Therefore, the effect of WM load on modulation of the N2 component in individuals with HTA was of particular interest in the present study.

Based on previous behavioral findings [21], we hypothesized that increasing WM load would delay RTs for incongruent trials, but would have no effect on congruent trials. Correspondingly, at the electrophysiological level, we also expected to find larger N2 amplitudes for incongruent trials when WM load was high. No study has investigated the cognitive load effects on conflict-related N2 components using a dual-task design that controls WM load. Therefore, the present results will provide novel insights into the neural correlations of the effect of WM load on conflict processing.

Our key prediction concerns the effect of trait anxiety on flanker interference effects under WM load. We focused on the interference effects on RTs and N2 amplitudes between two groups under different WM loads. Under high WM load, based on the ACT, we expected that relative HTA individuals would exhibit larger interference effects than LTA individuals on RTs and N2 amplitudes, which is suggestive of less efficient inhibition of task-irrelevant distractors in HTA individuals than in LTA individuals. Under low WM load, we made two predictions based on the ACT and previous works [8]. Based on the ACT, if HTA individuals show a compensatory strategy of recruiting greater cognitive resources to achieve task performance comparable to that of LTA individuals, they would display comparable interference effects on RTs but smaller interference effects on N2 amplitudes. However, Pacheco-Unguetti et al. [8] found that HTA participants had greater difficulties than LTA participants in controlling interference from the flanker task, in which WM load was not manipulated. Therefore, based on the finding of Pacheco-Unguetti et al. [8], we considered the other possibility that under low WM load, HTA individuals would show larger interference effects on RTs and N2 amplitudes compared with LTA individuals. Furthermore, such group differences in interference effects would be smaller than those under high WM load.

## Method

### Ethics Statement

This study was approved by the ethics committee of Shaanxi Normal University of China. The study adhered to the guidelines as set out in the Declaration of Helsinki. All participants gave written consent after the procedures were explained and were debriefed after the experiment. Participants were paid 45 RMB for their time.

### Participants

Initially, 1240 undergraduate students from Shaanxi Normal University, China, took part in a mass screening using the Chinese version of the trait anxiety portion of Spielberger's State-Trait Anxiety Inventory [39], [40], which was completed as part of a pre-test. Subsequently, participants who scored high in trait anxiety (HTA group; upper 27th percentile of the distribution) or had low levels of trait anxiety (LTA group; lower 27th percentile of the distribution) were chosen for further consideration. From these groups, we invited 19 female HTA participants (mean age of 19.89 years) and 18 female LTA participants (mean age of 20.06 years) to take part in the study. Only female participants were chosen in order to control for the gender differences in cognitive control [41]. Additionally, because most classes in Shaanxi Normal University are dominated by female students, not enough male participants in the mass screening could be recruited for a balanced gender distribution in our sample. All of the participants were tested within two weeks of their pre-test. Before the EEG testing session, each participant provided demographic information (Table 1) and was reassessed with the Trait Anxiety Inventory (post-test). Descriptives for trait anxiety scores of 37 participants are shown in Table S1. An independent-samples *t*-test revealed that the HTA group had higher trait anxiety scores than the LTA group in the pre-test, *t*(35) = 18.03, *p*<.001, and the post-test, *t*(35) = 13.01, *p*<.001. All participants reported not being regular users of medication or other nonmedical substances that can potentially affect the central nervous system. All participants reported being right-handed with normal or corrected-to-normal vision as well as having no history of psychiatric or neurological disorders.

**Table 1:** Demographic information for the high-trait-anxious (HTA) and low-trait-anxious (LTA) groups (Mean ± Standard deviation).

Group	Pre-test, TA score	Post-test, TA score	BDI score	Age
HTA	58.95 (5.59)	56.58 (7.10)	15.89 (3.68)	19.89 (1.41)
LTA	29.56 (4.18)	30.39 (4.88)	4.67 (4.44)	20.06 (1.51)

TA  =  Trait anxiety; BDI  =  Beck depression inventory.

### Stimuli and Procedure

In an electromagnetically shielded room, participants were seated comfortably about 70 cm away from a 19-in. screen. The participants performed a dual-task with a WM task (letter recall) and a flanker task (arrow identification). In the WM task, WM load was manipulated by varying the memory set size with either one letter (low WM load) or six letters (high WM load). These consonant letter strings were created using a random letter generator (http://www.dave-reed.com/Nifty/randSeq.html). In the flanker task, a central arrow (1.48°×0.82°) was flanked by two distractor arrows. The distance between the arrows was 0.16°. Distractor arrows were pointed either at the same direction (i.e., congruent trial) or at opposite directions (i.e., incongruent trial) as the central target arrow. An equal probability was set for each flanker trial being congruent or incongruent.

**Figure 1:**
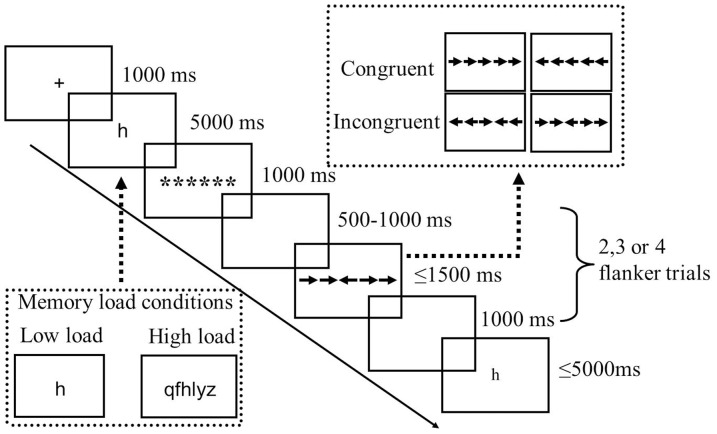
Procedure used in the present experiment, showing an incongruent trial under low working memory load.

Figure 1 depicts a sample trial sequence of the task. All stimuli were presented in black on a gray background. A fixation cross was displayed for 1000 ms, followed by a memory set for 5000 ms. A masking array with a row of six asterisks (7.58°×1.23°) was then presented for 1000 ms, allowing the participants to have an equal spatial attention window after different letter strings. After a randomized blank screen for 500–1000 ms, the arrow flanker task was presented for 1500 ms or until a response was obtained. In this task, the random blank screen was chosen to discourage participants from adopting a strategy to predict the onset of the next flanker task. In addition, the random interval could make the pre-stimulus ERP baseline smooth and steady. Participants were required to respond as quickly and accurately as possible to the direction of the central arrow (left or right) by using their right hand to press “1” on the numeric keypad if the central arrow pointed to the left, or “2” if it pointed to the right. To ensure that the memory set was actively rehearsed during the entire trial, the presentation of the memory probe was made unpredictable by varying the number of flanker trials (two, three, or four trials) [42]. After a sequence of flanker trials, a single-letter memory probe was presented for 5000 ms or until a response was obtained. Subjects were asked to indicate whether the memory probe letter was present in the preceding memory set by using their left hand to press the “c” key if the memory probe letter was present or the “v” key if it was not. Key allocations were counterbalanced between participants. The probe letter was equally likely to have been present or absent in the memory set. The inter-trial interval was 2000 ms.

Our experiment was comprised of three runs. Each run included two blocks manipulated with high or low WM load, respectively. The two blocks were presented randomly. Each block has 24 WM trials and a total of 68 flanker trials. Overall, participants went through 144 WM trials and 408 flanker trials. A block design for the WM load manipulation was used because intermixing trials of different WM loads in one block would require switching between the different types of trials and would result in a general increase in cognitive control. Such increased cognitive control might reduce the potency of the manipulation of WM load [21]. Participants were instructed to maintain central fixation and encouraged to minimize eye blinks during EEG recording.

### Electrophysiological data recordings and measurement

Brain electrical activity was recorded at 64 scalp sites using tin electrodes mounted on an elastic cap (Brain Product, München, Germany), with references on the left and right mastoids, and a ground electrode on the medial frontal aspect. Vertical electrooculograms (EOGs) were recorded supra- and infra-orbitally at the left eye. The horizontal EOG was recorded from the left versus right orbital rim. The EEG and EOG were amplified using a 0.05- to 100- Hz bandpass and were continuously digitized at 500 Hz/channel. All inter-electrode impedances were maintained below 5 kΩ. The EEG data was processed and analyzed using Brain Vision Analyzer software (Brain Products). Offline, the data were referenced to the average of the left and right mastoids (average mastoid reference), and these signals were bandpass filtered off-line using phase shift-free Butterworth filters with half-power cutoffs at 0.1 and 30 Hz and a roll-off of 24 dB/octave. Trials containing saccades, blinks, or muscle artifacts (EOG voltage exceeding ±75 µV) and those contaminated with artifacts due to amplifier clipping and peak-to-peak deflection exceeding ±75 µV were excluded from the average. The percentages of trials excluded from the averaging were 13.4% for the LTA group and 12.5% for the HTA group because of saccades, blinks, or muscle artifacts. Only trials with correct flanker responses and correct memory test results were included for an average.

The averaged epoch for ERP was 700 ms, including 100 ms pre-flanker-stimuli and 600 ms post-flanker-stimuli onset. Separate averages were calculated for each participant as a function of both WM load (low, high) and congruency (congruent, incongruent). Correct-trial congruent and incongruent amplitudes for the N2 under conditions of low or high WM load were extracted as the average of 20 ms pre-peak to 20 ms post-peak negative amplitude between 250 ms and 380 ms at Fz, FCz, and Cz electrode sites. The N2 peak was defined as the local peak. These electrodes were chosen based on previous research demonstrating that the N2 is focal over fronto-central locations [33], [36]. Latency measurements for the N2 were indexed as the time of the peak negative-going amplitude within the same 250–380 ms window.

N2 amplitudes and latencies data were entered into separate 2×2×2×3 mixed analyses of variance (ANOVA), with group (HTA, LTA) as the between-subjects factor, and congruency (congruent, incongruent), WM load (low, high), and electrode (Fz, FCz, and Cz) as within-subjects factors. Appropriate Greenhouse-Geisser adjustments to the degrees of freedom were performed. Bonferroni corrections were used for each comparison. Partial-eta^2^ (η^2^_P_) is reported as a measure of effect size.

## Results

### Behavioral data

#### Working memory task

Descriptives of probe error rates in the WM task as a function of WM load can be found in Table S2. Accuracy, rather than speed, was emphasized to participants for the memory probe response. Therefore, only probe error rates were analyzed. Error rates were entered into a 2×2 mixed ANOVA with group (HTA, LTA) as the between-subjects factor and WM load (low, high) as the within-subjects factor. ANOVA yielded a significant main effect of WM load, *F*(1,35) = 71.89, *p*<0.001, η^2^_P_ = 0.673, which shows that probe error rates were higher under high WM load (*M* = 6.4%, *SD* = 3.0) than under low WM load (*M* = 1.7%, *SD* = 2.2). This result confirmed that the WM load manipulation was successful in the present study. However, neither the main effect of group, *F*(1,35) = 1.35, *p* = 0.253, η^2^_P_ = 0.037, nor the two-way interaction, *F*(1,35) = 0.66, *p* = 0.422, η^2^_P_ = 0.019, was significant, which indicates that WM load levels affected the memory task performance similarly in the two groups.

In addition, we confirmed that the result of error rates was the same for all numbers of flanker trials before memory-probe (two, three, or four flanker trials), given that neither the main effect of number, *F*(2,70) = 0.25, *p* = 0.76, η^2^_P_ = 0.007, nor the interactions with number were significant, ([*Fs*] <2.55, *p*s>0.09).

#### Flanker task

Descriptives of mean RTs and error rates in the flanker task as a function of WM load and congruency can be found in Table S3. For the RTs analysis of arrow identification responses, trials with responses that were incorrect or faster than 200 ms were excluded. In addition, considering that the prerequisite for investigating the effect of WM load on cognitive control was that participants should be able to remember memory items successfully, flanker trials were also excluded from the RTs analysis if the response to the memory probe after such flanker trials was incorrect. We initially checked whether the RTs and error rates in the flanker task vary as a function of the number of flanker trials before memory-probe (two, three, or four flanker trials). For the RTs, neither the main effect of number, *F*(2,70) = 0.56, *p* = 0.57, η^2^_P_ = 0.016, nor the interactions with number were significant ([*Fs*] <0.78, *p*s>0.43). For the error rates, neither the main effect of number, *F*(2,70) = 1.57, *p* = 0.22, η^2^_P_ = 0.043, nor the interactions with number were significant ([*Fs*] <2.58, *p*s>0.10). Therefore, the main analyses were averaged across these flanker trials. Table 2 depicts mean correct RTs and error rates for the two groups as a function of congruency and WM load.

**Table 2:** Mean reaction times (RTs) and error rates in the flanker task as a function of WM load, congruency types, and groups (Mean ± Standard deviation).

		Low WM load		High WM load	
		HTA	LTA	HTA	LTA
RTs (ms)	Congruent	523 (61)	513 (50)	524 (55)	524 (56)
	Incongruent	567 (62)	560 (54)	620 (51)	578 (69)
	Interference effect	43 (19)	47 (19)	96 (40)	54 (24)
Error rates (%)	Congruent	0.1 (0.2)	0.3 (0.6)	0.1 (0.3)	0.2 (0.5)
	Incongruent	0.8 (1.8)	0.9 (1.0)	0.9 (1.8)	0.7 (0.8)
	Interference effect	0.7 (1.8)	0.6 (0.9)	0.8 (1.8)	0.4 (1.0)

HTA  =  high-trait-anxious, LTA  =  low-trait-anxious; Interference effect  =  incongruent minus congruent.

Mean correct RTs were entered into 2×2×2 mixed ANOVA, with group (HTA, LTA) as the between-subjects factor, and congruency (congruent, incongruent) and WM load (low, high) as within-subjects factors. ANOVA yielded a significant main effect of congruency, *F*(1,35) = 287.15, *p*<0.001, η^2^_P_ = 0.891, which indicates faster RTs for congruent trials (*M* = 521 ms, *SD* = 54) than for incongruent trials (*M* = 581 ms, *SD* = 57). This main effect was qualified by obtaining a significant two-way interaction between congruency and WM load, *F*(1,35) = 31.28, *p*<0.001, η^2^_P_ = 0.472. Follow-up analyses revealed that the RT interference effect (i.e., incongruent minus congruent) was larger under high WM load (*M* = 76 ms, *SD* = 39) than under low WM load (*M* = 45 ms, *SD* = 18), *t*(36) = 4.70, *p*<0.001, indicating that the increase in WM load exacerbated the interference effect on collapsed RTs across groups. This effect was primarily driven by the higher RTs for incongruent trials under high WM load compared to those under low WM load, *t*(36) = 6.91, *p*<0.001. Meanwhile, no significant difference was found between the two WM loads in congruent trials, *t*(36) = 1.92, *p* = 0.063.

**Figure 2:**
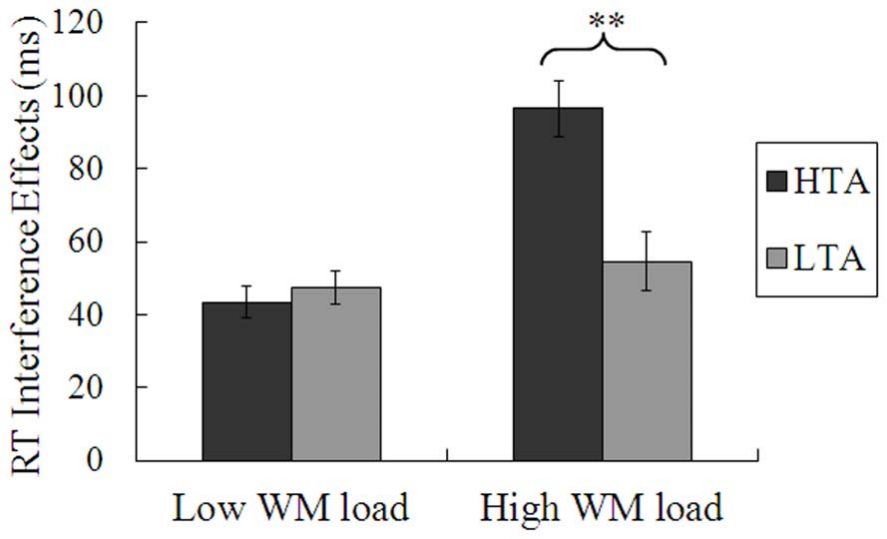
Interference effects (i.e., incongruent-congruent) on mean reaction times (RTs) under low or high working memory (WM) loads, for the high-trait-anxious (HTA) and low-trait-anxious (LTA) groups. Error bars represent standard errors of the means. **p< .01.

More importantly, the three-way interaction between congruency, WM load, and group was significant, *F*(1,35) = 18.14, *p*<0.001, η^2^_P_ = 0.341. An examination of the interference effect on RTs within each WM load revealed a group × congruency interaction under high WM load, *F*(1,35) = 14.37, *p* = 0.001, η^2^_P_ = 0.291, but not under low WM load, *F*(1,35) = 0.44, *p* = 0.51, η^2^_P_ = 0.012. When WM load was low, the interference effect on RTs (see Figure 2) did not differ between the HTA group (*M* = 43 ms, *SD* = 19) and the LTA group (*M* = 47 ms, *SD* = 19), *t*(35) = 0.66, *p* = 0.51. On the contrary, when WM load was high, the interference effect on RTs was greater in the HTA group (*M* = 96 ms, *SD* = 40) than in the LTA group (*M* = 54 ms, *SD* = 24), *t*(35) = 3.79, *p* = 0.001. This effect was driven by higher RTs for incongruent trials in the HTA group than in the LTA group, *t*(35) = 2.11, *p* = 0.042. No significant difference was observed between the two groups in congruent trials, *t*(35) = 0.01, *p* = 0.99.

Error rates were analyzed in the same way as RTs. For error rates, mixed ANOVA revealed a main effect of congruency, *F*(1,35) = 9.90, *p* = 0.003, η^2^_P_ = 0.22, which shows that participants committed more errors in incongruent trials (*M* = 0.9%, *SD* = 1.3) than in congruent trials (*M* = 0.2%, *SD* = 0.3). The main effects of WM load, group, and all remaining interactions were not significant (all *p*>0.21).

### ERP data

**Figure 3:**
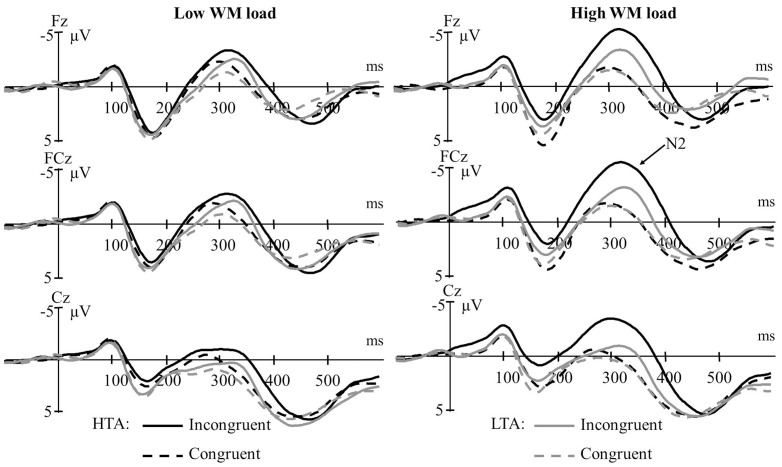
Grand average waveforms for the high-trait-anxious (HTA) and low-trait-anxious (LTA) groups in each condition over Fz, FCz, and Cz sites.

Figure 3 depicts the grand average waveforms for each group in each condition. For the LTA group, ERPs contained an average ± standard deviation of 87±6 trials for congruent trials under low WM load, 85±7 for incongruent trials under low WM load, 87±6 for congruent trials under high WM load, and 86±6 for incongruent trials under high WM load. For the HTA group, ERPs contained an average of 89±3 trials for congruent trials under low WM load, 86±5 for incongruent trials under low WM load, 89±3 for congruent trials under high WM load, and 85±6 for incongruent trials under high WM load. Notably, noise estimates were found to be similar between groups ([*t*s] <1.16, *p*s>0.25), therefore suggesting that noise did not differentially bias amplitude measurement for either group [43].

#### N2 amplitude

Descriptives of N2 amplitudes data recorded from three electrodes can be found in Table S4. Table 3 shows the mean amplitudes of N2 on the flanker task as a function of conditions for the HTA and LTA groups is showed in. ANOVA yielded a significant main effect of electrode, *F*(2,70) = 17.55, *p*<0.001, η^2^_P_ = 0.334, which shows that N2 amplitudes were larger at both Fz and FCz than at Cz (all comparisons *p*<0.005), but did not differ between Fz and FCz. The interaction between congruency and WM load was significant, *F*(1,35) = 61.94, *p*<0.001, η^2^_P_ = 0.639. Follow-up analyses showed that the modulation of N2 amplitudes through WM load was only observed for incongruent trials (*p*<0.001), but not for congruent trials (*p* = 0.77). The interference effect on N2 amplitudes was significantly larger under high WM load (*M* = −2.47 µV, *SD* = 1.28) than that under low WM load (*M* = −1.20 µV, *SD* = 0.91), *t*(36) = −5.98, *p*<0.001.

**Table 3:** N2 amplitudes (µV) in the flanker task as a function of conditions for the high-trait-anxious (HTA) and low-trait-anxious (LTA) groups (Mean ± Standard deviation).

		Low WM load		High WM load	
		HTA	LTA	HTA	LTA
Fz	Congruent	−2.61 (3.13)	−2.17 (3.13)	−1.80 (3.24)	−2.42 (3.45)
	Incongruent	−3.86 (3.31)	−3.38 (2.67)	−5.74 (3.21)	−4.13 (3.43)
FCz	Congruent	−2.22 (2.98)	−1.86 (3.42)	−1.90 (3.02)	−2.41 (3.56)
	Incongruent	−3.68 (2.83)	−3.08 (3.21)	−5.89 (2.70)	−3.90 (3.61)
Cz	Congruent	−0.82 (3.09)	0.05 (3.79)	−0.66 (3.96)	−0.90 (4.23)
	Incongruent	−2.17 (3.30)	−0.63 (4.03)	−3.15 (3.43)	−1.96 (3.88)

**Figure 4:**
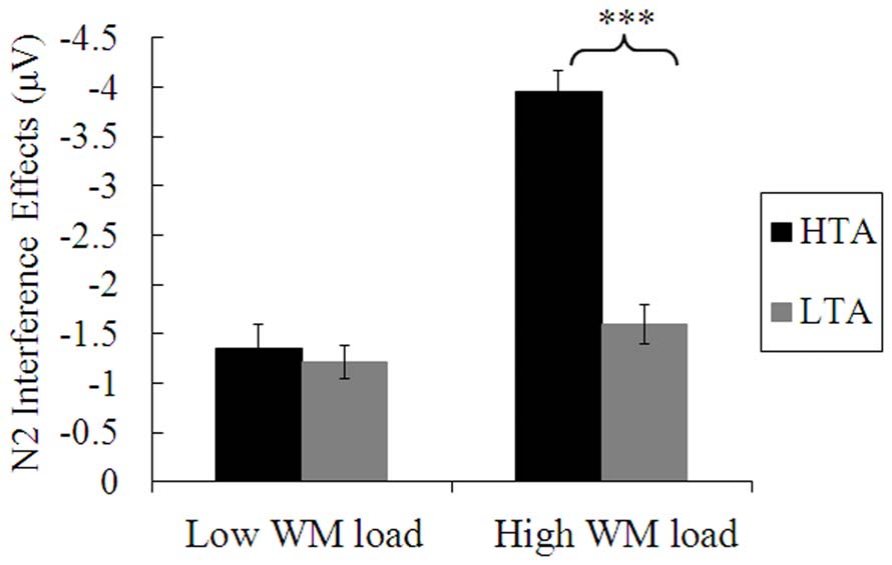
Interference effects (i.e., incongruent-congruent) on N2 amplitudes under low and high working memory (WM) loads, for the high-trait-anxious (HTA) and low-trait-anxious (LTA) groups. Error bars represent standard errors of the means. ***p< .001.

Moreover, a significant interaction was observed between electrode and congruency, *F*(2,70) = 11.03, *p*<0.001, η^2^_P_ = 0.24, which shows that the interference effect on N2 amplitudes was larger at Fz and FCz than at Cz (all comparisons *p*<0.001), but did not differ between Fz and FCz (*p = *0.89). Critically, a significant four-way interaction among electrode, congruency, WM load, and group was observed, *F*(2,70) = 3.85, *p* = 0.046, η^2^_P_ = 0.099. Follow-up analyses showed that the interaction of congruency, WM load, and group was significant at Fz, *F*(1,35) = 22.47, *p*<0.001, η^2^_P_ = 0.391, and FCz, *F*(1,35) = 55.45, *p*<0.001, η^2^_P_ = 0.613, but not at Cz, *F*(1,35) = 1.57, *p* = 0.218, η^2^_P_ = 0.043, which indicates that WM load modulated the interaction between group and congruency at both Fz and FCz. Therefore, an examination of the interaction between group and congruency within each WM load for average N2 amplitudes across two electrodes (Fz and FCz), revealed a group × congruency interaction under high WM load, *F*(1,35) = 67.53, *p*<0.001, η^2^_P_ = 0.659, but not under low WM load, *F*(1,35) = 0.21, *p* = 0.66, η^2^_P_ = 0.006. When WM load was low, the interference effect on N2 amplitudes (see Figure 4) did not vary between the HTA group (*M* = −1.35 µV, *SD* = 1.08) and the LTA group (*M* = −1.22 µV, *SD* = 0.71), *t*(35) = −0.45, *p* = 0.66. By contrast, when WM load was high, the interference effect on N2 amplitudes was significantly larger for the HTA group (*M* = −3.96 µV, *SD* = 0.91) than for the LTA group (*M* = −1.60 µV, *SD* = 0.84), *t*(35) = −8.22, *p*<0.001.

#### N2 latency

Descriptives of N2 latencies data recorded from three electrodes can be found in Table S5. ANOVA revealed a main effect of congruency, *F*(1,35) = 12.65, *p* = 0.001, η^2^_P_ = 0.265, which shows that the time to peak latencies for the N2 was earlier in congruent trials (*M* = 306 ms, *SD* = 35) than in incongruent trials (*M* = 314 ms, *SD* = 34). A significant main effect of electrode, *F*(2,70) = 13.61, *p*<0.001, η^2^_P_ = 0.28, was also observed, showing that the time to peak latencies for the N2 was later at Fz than at FCz and Cz (all comparisons *p*<0.007). However, the main effects of WM load, group and all other interactions were non-significant for the time to peak latencies for the N2 (all *p*>0.34).

### Correlational analyses

**Figure 5:**
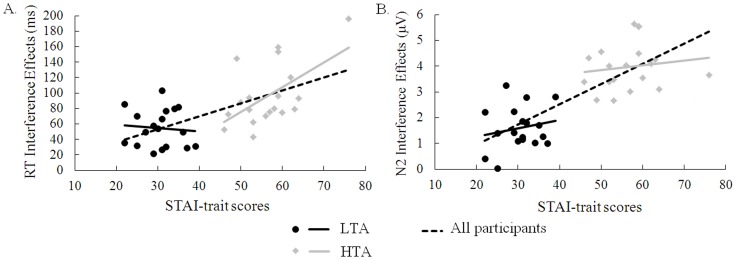
Scatterplots of the relationship between trait anxiety (TA) scores and interference effects (i.e., incongruent-congruent) on reaction times (RTs) (A) and N2 mean amplitudes (B) under high WM load. STAI  =  State-Trait Anxiety Inventory. Note that here the inverse of N2 amplitude data was used for correlations.

To examine the relationship between trait-anxiety (TA) scores and indexes of interference effect (calculated by subtracting congruent trials from incongruent trials for RTs, N2 amplitudes) under low or high WM load, we first included all participants in the correlational analyses. The post-test scores of TA and the averaged N2 amplitudes across two electrodes (Fz and FCz) were used for correlational analyses. Fz and FCz were selected because the maximal interference effect on N2 amplitudes was found in these two electrodes. For ease of interpretation, the inverse of N2 amplitude data was used for correlations so that positive correlations reflected increased interference effects similar to those of RTs. Table 4 shows the correlation coefficients. Higher TA scores were associated with larger mean RT interference effect under high WM load, Pearson's *r*(37) = 0.63, *p*<0.001 (see Figure 5A), but not under low WM load, *r*(37) = −0.02, *p* = 0.90. Higher TA scores were associated with larger N2 interference effect under high WM load, *r*(37) = 0.78, *p*<0.001(see Figure 5B), but not under low WM load, *r*(37) = 0.18, *p* = 0.28. Therefore, higher levels of trait anxiety were associated with increased behavioral and neural indexes of interference effect. In addition, the correlation between RT interference effect and N2 interference effect was significant under high WM load, *r*(37) = 0.55, *p*<0.001, but not under low WM load, *r*(37) = −0.04, *p* = 0.80.

**Table 4:** Correlations between trait anxiety (TA) scores and indices of interference effects (i.e., incongruent-congruent) on reaction responses (RTs) and N2 amplitudes under low or high WM load.

Group		1	2	3	4	5
All Participants	1. TA scores	-				
	2. Low load-RTs	−0.02	-			
	3. High load-RTs	0.63**	0.21	-		
	4. Low load-N2	0.18	−0.04	−.12	-	
	5. High load-N2	0.78**	−0.08	0.55**	0.23*	-
HTA group	1. TA scores	-				
	2. Low load-RTs	0.36	-			
	3. High load-RTs	0.57*	0.10	-		
	4. Low load-N2	0.23	0.01	−0.27	-	
	5. High load-N2	0.15	0.09	0.27	0.11	-
LTA group	1. TA scores	-				
	2. Low load-RTs	−0.06	-			
	3. High load-RTs	−0.09	0.74**	-		
	4. Low load-N2	0.37	−0.10	0.01	-	
	5. High load-N2	0.20	−0.07	0.13	0.64**	-

*p<.05, **p<.01; HTA  =  high-trait-anxious, LTA  =  low-trait-anxious; Note that here the inverse of N2 amplitude data was used for correlations.

Second, we examined the correlations between TA scores and interference effect indexes under low or high WM load separately for the HTA group and the LTA group. Under high WM load, higher TA scores were associated with larger mean RT interference effect for the HTA group, *r*(19) = 0.57, *p* = 0.01, but not for the LTA group, *r*(18) = −0.09, *p* = 0.73 (see Figure 5A). This finding suggests that this relationship is specific to HTA individuals rather than a function of general individual differences in anxiety. Additionally, in the LTA group, the correlation between RT interference effect under high WM load and RT interference effect under low WM load, *r*(18) = 0.74, *p*<0.001, and the correlation between N2 interference effect under high WM load and N2 interference effect under low WM load, *r*(18) = 0.64, *p* = 0.004, were both significant. However, none of the remaining correlations were significant ([*rs*] <0.37, *ps*>0.12; see Table 4).

## Discussion

The present study is the first to utilize a dual-task design using an electrophysiological measure to investigate whether and how WM load modulates cognitive control of flanker distractors in trait anxiety. First, incongruent flanker stimuli were more difficult to inhibit under higher WM loads, as indicated by increased RTs and more negative N2 amplitudes in incongruent trials under high WM load than under low WM load. Second, a major finding of the present study is that the association between trait anxiety and the inhibition of distractors varies as a function of WM load. Specifically, HTA individuals experienced greater interference from irrelevant distractors than did LTA individuals under high WM load, but not under low WM load. These findings suggest that trait anxiety is associated with deficits of inhibiting task-irrelevant distractors only in situations where limited WM resources are depleted by high WM load.

Our first result replicated those of Lavie et al. [21]. Specifically, the results showed that high WM-related cognitive demand reduced the ability of all participants to inhibit flanker distractors at the behavioral level. Extending previous research [21], we observed more negative N2 amplitudes in incongruent trials under high WM load than in low WM load. Larger N2 is also associated with a higher degree of current trial conflict [34], [36], [37], and the neural signal indexed by N2 is influenced by the adjustment of cognitive control [33], [36]. Moreover, previous studies have suggested that compromised attentional control results in more negative N2 amplitudes for incongruent trials [33], [35]. Consequently, our finding of WM load modulation of the N2 amplitudes indicates that when limited resources are consumed by the concurrent high load task, less allocation of WM resources to inhibit distractors causes a greater degree of response conflict. This explanation is consistent with the idea that WM and cognitive control rely on the same resources [24], [29].

More importantly, HTA and LTA groups differed in their abilities to inhibit distractors when limited WM resources were consumed by high WM load. Specifically, when the WM load was high, the HTA group took a longer time than the LTA group to respond in incongruent trials, where participants required top-down control of attention to inhibit flanker distractors. On the contrary, when the WM load was low, the HTA group did not perform worse on congruent trials, where inhibition was not required. Furthermore, the increased RTs interference effect for HTA individuals was accompanied through a modulation of the N2 amplitudes, which is reflective of an increased degree of response conflict for HTA individuals. Based on previous studies [33], [35], the increment in the N2 interference effect for HTA individuals was likely a result of their compromised conflict control under high WM load. Therefore, our findings suggest that under high WM load, HTA individuals exhibit less efficient recruitment of the top-down mechanisms required for conflict control, therefore resulting in greater conflict signal.

Our findings under high WM load are analogous to those of Sadeh and Bredemeier [18]. Both studies indicate an impaired inhibition of irrelevant distractors in HTA individuals when attentional resources are fully occupied by tasks. Sadeh and Bredemeier [18] also found that the impairment in the HTA group was specific to high response conflict under high perceptual load rather than under low perceptual load. In the present study, such impairment in the HTA group was also observed under high WM load but not under low WM load. Therefore, a critical difference between the two studies lies in the different types of load manipulated in the tasks. Specifically, the higher perceptual capacity in HTA individuals allows them to perceive both task-relevant and task-irrelevant information under higher levels of perceptual load [18]. On the contrary, the increased perception of irrelevant distractors in HTA individuals under higher levels of WM load might be due to their reduced WM capacity, as shown in previous studies [44]. This explanation is consistent with the idea that individuals with low WM capacity are more likely to be affected by irrelevant information than those with high WM capacity [45], [46]. Taken together, although different types of load and processing capacity are involved, the impaired inhibition of distractors in HTA individuals was observed in both studies when active attentional control was required to inhibit the processing of irrelevant distractors and when tasks became more demanding in terms of attention and execution control.

Furthermore, impaired inhibition in trait anxiety was also quantified by our correlation analyses. The correlations for all participants showed that higher levels of trait anxiety were associated with larger behavioral and neural interference effect indexes, respectively. In an anti-saccade task, Berggren et al. [32] found a similar significant correlation between trait anxiety scores and the RT differences between anti-saccade and pro-saccade trials under high WM load, but not under low WM load. However, our results under high WM load clearly indicated that trait anxiety impaired performance in the flanker task, but not in the WM task, therefore excluding the possibility that WM load effects on anxiety may have impaired performance on both tasks. Moreover, our findings also extend the behavioral data of Berggren et al. [32] by revealing abnormal conflict processing in anxious individuals at the neural level.

Our findings of increased detriments to performance under high WM load in HTA individuals support the ACT's prediction [5], [11]. The ACT proposes that anxious individuals would show impaired cognitive control when tasks place relatively high demand on cognitive resources, because the compensatory strategy of HTA individuals may be disrupted by high task demands. With the imposition of high WM load on cognitive control in the present task, the compensatory strategies of HTA participants might have been disrupted, considering that WM resources available for subsequent active control were increasingly strained. This effect is likely the reason why HTA individuals were affected more by response-competing distractors than LTA participants.

Under low WM load, HTA individuals exhibit the same ability to inhibit task-irrelevant information as LTA individuals in either RTs or N2 amplitudes. These findings are in contrast with our prediction. Based on the ACT, we predicted that HTA and LTA participants would exhibit comparable RTs for incongruent trials, but that HTA participants would show reduced N2 amplitudes for incongruent trials. This result would mean that HTA individuals could use a compensatory strategy to achieve task performance that is comparable with that of LTA individuals by utilizing greater cognitive resources. However, our data showed that HTA individuals did not exhibit disrupted cognitive control in their behavioral performance. We also failed to find evidence of reduced N2 amplitudes for incongruent trials in HTA individuals, which would be indicative of a compensatory process [35]. Therefore, the present findings under low WM load does not support the prediction of the ACT mentioned above, but indicate that trait anxiety is not linked to a deficit in inhibition of task-irrelevant information when a low WM load is imposed on cognitive control processes.

In addition, our findings under low WM load appear to be in contrast with those of Pacheco-Unguetti et al. [8], who suggested that HTA participants had greater difficulties than LTA participants in controlling flanker interference. In their task, the researchers combined a flanker paradigm with a spatial cueing procedure, in which participants performed a flanker task before an alerting task and an orienting task. Task demands for WM resources may have been relatively high in these tasks. Therefore, in line with our findings under high WM load, Pacheco-Unguetti et al. [8] found that trait anxiety was related to a deficit in inhibition control. Moreover, a previous study has indicated that alertness impairs inhibition control in flanker tasks [47]. Hence, alertness possibly hampers performance to a greater extent in HTA individuals with pre-existing deficits in inhibition control than in LTA individuals.

Our findings under high and low WM load could provide a potential explanation for previously inconsistent results. Certain authors found that trait anxiety was associated with a deficit in inhibition [7], [8], [10], whereas others did not find such evidence [15]–[17]. The present results therefore suggest considering WM-related cognitive demand involved in tasks, as it was by manipulating WM load that the differences between HTA and LTA individuals in cognitive control were observed in this study. Our results indicate that a high WM tax on cognitive control processes is sufficient to induce inhibition control deficits in anxious individuals. Therefore, the lack of evidence of cognitive deficits in trait anxiety, which were reported in previous studies, might be attributed to the relatively low WM demands in those tasks. However, when task demands on WM resources are high, HTA individuals are less efficient in the inhibition of irrelevant information. Therefore, manipulations of WM-related cognitive demand are important for elucidating cognitive control deficits in trait anxiety.

Speculating which neural circuits underlie the observed behavioral and ERP results is tempting. Previous research has consistently demonstrated that both dorsolateral PFC and dorsal ACC affected the detection of conflict and cognitive control implementation in flanker tasks [30], [31]. At the same time, the PFC during the delay-period of a WM task is believed to reflect “top-down” maintenance of stimuli representations at lower sensory levels, and such maintenance inherently involves the inhibition of competing representations [24]. Furthermore, high WM load activates greater dorsolateral PFC than low WM load [20], [29]. Therefore, when limited WM resources are depleted by high WM load, the larger interference effects in RTs and N2 in HTA individuals are likely caused by the lesser activation of dorsolateral PFC involved in inhibiting distractor processing, therefore leading to a greater activation of dorsal ACC. Future studies should examine this possibility directly in HTA individuals using fMRI.

The current results should also be considered within the context of several strengths and limitations. First, only female participants were recruited to maintain group homogeneity. However, given the existence of sex differences in the susceptibility to emotional disorders, future studies should further clarify possible sex differences in the observed effects. Second, Ahmed and de Fockert [42] identified an effect of WM capacity on distractibility using a very similar task. Hence, given the risk for a confounding variable of WM capacity in our study and its potential relationship with trait anxiety, the WM capacity of individuals should be an important aspect to assess. Third, the present study did not consider the influence of depression. However, given the comorbidity between anxiety and depression, future research should examine the independent and interactive effects of anxiety and depression in relation to cognitive control under different WM loads. Individuals with high or low trait-anxiety are typically used in studies that investigate the ACT. Fourth, an engineered response box is preferable to keyboard buttons for measuring RTs because the response box features a 0 millisecond de-bounce period, which cannot be achieved from a standard keyboard. Although we have achieved meaningful results with a keyboard, future research should use an engineered response to measure RTs more accurately.

Extending previous research, our findings support placing a high WM load on tasks as a contextual factor that engenders cognitive control deficits in anxious individuals. When HTA individuals are concurrently taxed by a high memory load, they become less efficient at using the top-down mechanisms required for inhibiting distractors. Our findings have a practical implication for teaching designs for anxious individuals. In particular, ensuring that teaching tasks carry low WM demands will be useful in preventing anxious individuals from distracting stimuli.

## Supporting Information

Table S1 **Demographic information for 37 participants.**
(DOC)

Table S2 **Probe error rates in the working memory task for 37 participants as a function of working memory load.**
(DOC)

Table S3 **Mean reaction times (RTs) and error rates in the flanker task for 37 participants as a function of working memory load and congruency.**
(DOC)

Table S4 **N2 amplitudes ( µV) data recorded from three electrodes in the experiment.**
(DOC)

Table S5 **N2 latencies (ms) data recorded from three electrodes in the experiment.**
(DOC)
