# Supplementary material for: Impact of Working Memory Load on Cognitive Control in Trait Anxiety: An ERP Study
Source: PLoS One. 2014 Nov 4;9(11):e111791. doi: 10.1371/journal.pone.0111791 (PMC4219777; doi:10.1371/journal.pone.0111791)
Supplement: Table S1 — Demographic information for 37 participants. (DOC) [file pone.0111791.s001.doc]

Table S1. Demographic information for 37 participants.

| Subject No. | Group | Age | Pre-test, TA scores | Post-test, TA scores | Depression scores |
| --- | --- | --- | --- | --- | --- |
| 1 | High-trait-anxious | 19 | 67 | 57 | 21 |
| 2 | High-trait-anxious | 23 | 70 | 76 | 18 |
| 3 | High-trait-anxious | 21 | 57 | 50 | 13 |
| 4 | High-trait-anxious | 21 | 67 | 60 | 19 |
| 5 | High-trait-anxious | 19 | 59 | 63 | 15 |
| 6 | High-trait-anxious | 18 | 55 | 62 | 14 |
| 7 | High-trait-anxious | 21 | 65 | 52 | 9 |
| 8 | High-trait-anxious | 19 | 54 | 52 | 15 |
| 9 | High-trait-anxious | 20 | 58 | 47 | 13 |
| 10 | High-trait-anxious | 22 | 60 | 64 | 13 |
| 11 | High-trait-anxious | 19 | 49 | 59 | 20 |
| 12 | High-trait-anxious | 20 | 58 | 59 | 12 |
| 13 | High-trait-anxious | 20 | 57 | 53 | 24 |
| 14 | High-trait-anxious | 19 | 62 | 58 | 13 |
| 15 | High-trait-anxious | 18 | 55 | 46 | 15 |
| 16 | High-trait-anxious | 21 | 63 | 59 | 14 |
| 17 | High-trait-anxious | 19 | 52 | 49 | 19 |
| 18 | High-trait-anxious | 21 | 53 | 53 | 17 |
| 19 | High-trait-anxious | 18 | 59 | 56 | 18 |
| 1 | Low-trait-anxious | 20 | 29 | 32 | 8 |
| 2 | Low-trait-anxious | 21 | 32 | 31 | 9 |
| 3 | Low-trait-anxious | 21 | 30 | 27 | 2 |
| 4 | Low-trait-anxious | 19 | 36 | 36 | 1 |
| 5 | Low-trait-anxious | 18 | 29 | 39 | 5 |
| 6 | Low-trait-anxious | 19 | 23 | 25 | 1 |
| 7 | Low-trait-anxious | 22 | 29 | 30 | 3 |
| 8 | Low-trait-anxious | 20 | 32 | 31 | 1 |
| 9 | Low-trait-anxious | 23 | 35 | 31 | 3 |
| 10 | Low-trait-anxious | 19 | 23 | 32 | 1 |
| 11 | Low-trait-anxious | 19 | 22 | 22 | 5 |
| 12 | Low-trait-anxious | 18 | 25 | 29 | 2 |
| 13 | Low-trait-anxious | 20 | 31 | 29 | 8 |
| 14 | Low-trait-anxious | 21 | 31 | 25 | 3 |
| 15 | Low-trait-anxious | 19 | 31 | 35 | 4 |
| 16 | Low-trait-anxious | 23 | 27 | 22 | 7 |
| 17 | Low-trait-anxious | 20 | 32 | 34 | 2 |
| 18 | Low-trait-anxious | 19 | 35 | 37 | 19 |
