# Supplementary material for: Impact of Working Memory Load on Cognitive Control in Trait Anxiety: An ERP Study
Source: PLoS One. 2014 Nov 4;9(11):e111791. doi: 10.1371/journal.pone.0111791 (PMC4219777; doi:10.1371/journal.pone.0111791)
Supplement: Table S2 — Probe error rates in the working memory task for 37 participants as a function of working memory load. (DOC) [file pone.0111791.s002.doc]

Table S2. Probe error rates in the working memory task for 37 participants as a function of working memory load.

| Subject No. | Group | Low load | High Load |
| --- | --- | --- | --- |
| 1 | High-trait-anxious | 0.03 | 0.05 |
| 2 | High-trait-anxious | 0 | 0.06 |
| 3 | High-trait-anxious | 0.06 | 0.08 |
| 4 | High-trait-anxious | 0 | 0.08 |
| 5 | High-trait-anxious | 0.01 | 0.06 |
| 6 | High-trait-anxious | 0 | 0.02 |
| 7 | High-trait-anxious | 0 | 0.04 |
| 8 | High-trait-anxious | 0 | 0.08 |
| 9 | High-trait-anxious | 0.01 | 0.03 |
| 10 | High-trait-anxious | 0.01 | 0.11 |
| 11 | High-trait-anxious | 0 | 0.02 |
| 12 | High-trait-anxious | 0.01 | 0.07 |
| 13 | High-trait-anxious | 0.05 | 0.08 |
| 14 | High-trait-anxious | 0.01 | 0.06 |
| 15 | High-trait-anxious | 0 | 0.05 |
| 16 | High-trait-anxious | 0.1 | 0.09 |
| 17 | High-trait-anxious | 0 | 0.08 |
| 18 | High-trait-anxious | 0 | 0.04 |
| 19 | High-trait-anxious | 0.01 | 0.01 |
| 1 | Low-trait-anxious | 0.02 | 0.07 |
| 2 | Low-trait-anxious | 0 | 0.1 |
| 3 | Low-trait-anxious | 0.02 | 0.07 |
| 4 | Low-trait-anxious | 0 | 0.09 |
| 5 | Low-trait-anxious | 0.04 | 0.06 |
| 6 | Low-trait-anxious | 0 | 0.05 |
| 7 | Low-trait-anxious | 0.01 | 0.09 |
| 8 | Low-trait-anxious | 0.02 | 0.07 |
| 9 | Low-trait-anxious | 0.01 | 0.05 |
| 10 | Low-trait-anxious | 0 | 0.02 |
| 11 | Low-trait-anxious | 0.02 | 0.13 |
| 12 | Low-trait-anxious | 0.03 | 0.11 |
| 13 | Low-trait-anxious | 0.04 | 0.02 |
| 14 | Low-trait-anxious | 0.01 | 0.03 |
| 15 | Low-trait-anxious | 0.03 | 0.07 |
| 16 | Low-trait-anxious | 0.06 | 0.05 |
| 17 | Low-trait-anxious | 0.02 | 0.07 |
| 18 | Low-trait-anxious | 0.01 | 0.12 |
