# Supplementary material for: Impact of Working Memory Load on Cognitive Control in Trait Anxiety: An ERP Study
Source: PLoS One. 2014 Nov 4;9(11):e111791. doi: 10.1371/journal.pone.0111791 (PMC4219777; doi:10.1371/journal.pone.0111791)
Supplement: Table S3 — Mean reaction times (RTs) and error rates in the flanker task for 37 participants as a function of working memory load and congruency. (DOC) [file pone.0111791.s003.doc]

Table S3. Mean reaction times (RTs) and error rates in the flanker task for 37 participants as a function of working memory load and congruency.

|  |  | Mean RTs | | | | Error rates | | |  |
| --- | --- | --- | --- | --- | --- | --- | --- | --- | --- |
| Subject No. | Group | Low load-congruent | Low load-incongruent | High load-congruent | High load-incongruent | Low load-congruent | Low load-incongruent | High load-congruent | High load-incongruent |
| 1 | High-trait-anxious | 595.9 | 643.4 | 590.1 | 665.81 | 0 | 0 | 0 | 0 |
| 2 | High-trait-anxious | 468.43 | 518.98 | 469.33 | 665.39 | 0 | 0 | 0 | 0.04 |
| 3 | High-trait-anxious | 504.57 | 564.89 | 525.5 | 613.44 | 0 | 0 | 0 | 0 |
| 4 | High-trait-anxious | 549.54 | 597.78 | 544.07 | 618.56 | 0 | 0 | 0 | 0 |
| 5 | High-trait-anxious | 566.4 | 612.58 | 558.62 | 637.83 | 0 | 0.01 | 0 | 0 |
| 6 | High-trait-anxious | 442.12 | 518.75 | 440.28 | 560.8 | 0 | 0.01 | 0 | 0.01 |
| 7 | High-trait-anxious | 518.31 | 570.25 | 507.84 | 586.33 | 0 | 0 | 0 | 0 |
| 8 | High-trait-anxious | 499.97 | 542.48 | 502.55 | 596.21 | 0 | 0.01 | 0 | 0 |
| 9 | High-trait-anxious | 433.54 | 488.4 | 447.78 | 520.12 | 0 | 0 | 0 | 0.01 |
| 10 | High-trait-anxious | 523.34 | 589.57 | 551.6 | 644.97 | 0 | 0 | 0 | 0 |
| 11 | High-trait-anxious | 461.5 | 492.21 | 463.75 | 617.38 | 0 | 0.07 | 0 | 0.07 |
| 12 | High-trait-anxious | 619.7 | 659.66 | 574.96 | 734.39 | 0 | 0 | 0 | 0.02 |
| 13 | High-trait-anxious | 479.29 | 522.06 | 510.59 | 572.75 | 0.01 | 0.02 | 0.01 | 0.02 |
| 14 | High-trait-anxious | 603.85 | 657.27 | 590.75 | 670.27 | 0 | 0 | 0 | 0 |
| 15 | High-trait-anxious | 567.78 | 563.92 | 584.24 | 636.57 | 0 | 0 | 0 | 0 |
| 16 | High-trait-anxious | 466.19 | 478.54 | 476.19 | 571.9 | 0 | 0.01 | 0.01 | 0 |
| 17 | High-trait-anxious | 469.02 | 493.03 | 452.82 | 597.35 | 0 | 0.01 | 0 | 0.01 |
| 18 | High-trait-anxious | 554.11 | 580.51 | 543.22 | 586.28 | 0 | 0 | 0 | 0 |
| 19 | High-trait-anxious | 618.08 | 671.02 | 620.19 | 690.26 | 0 | 0 | 0 | 0 |
| 1 | Low-trait-anxious | 483.99 | 546.18 | 484.71 | 561.24 | 0 | 0 | 0 | 0 |
| 2 | Low-trait-anxious | 518.6 | 574.73 | 512.69 | 579.22 | 0 | 0 | 0 | 0 |
| 3 | Low-trait-anxious | 477.4 | 526.2 | 466.07 | 515.92 | 0 | 0.01 | 0.01 | 0 |
| 4 | Low-trait-anxious | 514.59 | 572.28 | 520.62 | 570.31 | 0 | 0.01 | 0 | 0 |
| 5 | Low-trait-anxious | 539.78 | 579.17 | 545.91 | 577.04 | 0.01 | 0.01 | 0 | 0.01 |
| 6 | Low-trait-anxious | 515.11 | 577.3 | 526.25 | 596.82 | 0 | 0 | 0 | 0 |
| 7 | Low-trait-anxious | 506.4 | 584.25 | 568.36 | 622.29 | 0 | 0.02 | 0 | 0.01 |
| 8 | Low-trait-anxious | 537.68 | 613.68 | 562.53 | 666.17 | 0 | 0.01 | 0.01 | 0.03 |
| 9 | Low-trait-anxious | 490.16 | 525.55 | 482.42 | 509.46 | 0 | 0.01 | 0 | 0.02 |
| 10 | Low-trait-anxious | 512.59 | 520.53 | 520.64 | 551.31 | 0 | 0 | 0 | 0 |
| 11 | Low-trait-anxious | 603.62 | 657.04 | 615.61 | 701.6 | 0.01 | 0.04 | 0.02 | 0 |
| 12 | Low-trait-anxious | 505.29 | 555.6 | 520.13 | 577.8 | 0 | 0 | 0 | 0.01 |
| 13 | Low-trait-anxious | 442.91 | 469.53 | 466.37 | 488.42 | 0.02 | 0.01 | 0 | 0.01 |
| 14 | Low-trait-anxious | 465.98 | 500.97 | 497.58 | 529.33 | 0 | 0.01 | 0 | 0 |
| 15 | Low-trait-anxious | 624.82 | 663.97 | 653.08 | 734.93 | 0.01 | 0.01 | 0 | 0.01 |
| 16 | Low-trait-anxious | 451.93 | 485.33 | 450.44 | 486.01 | 0.01 | 0.02 | 0 | 0 |
| 17 | Low-trait-anxious | 460.6 | 527.67 | 457.18 | 536.72 | 0 | 0 | 0 | 0.01 |
| 18 | Low-trait-anxious | 575.26 | 600.5 | 576.84 | 605.92 | 0 | 0 | 0 | 0.01 |
|  | | | | | | | | | |
