# Supplementary material for: Impact of Working Memory Load on Cognitive Control in Trait Anxiety: An ERP Study
Source: PLoS One. 2014 Nov 4;9(11):e111791. doi: 10.1371/journal.pone.0111791 (PMC4219777; doi:10.1371/journal.pone.0111791)
Supplement: Table S4 — N2 amplitudes ( µV) data recorded from three electrodes in the experiment. (DOC) [file pone.0111791.s004.doc]

Table S4. N2 amplitudes (μV) data recorded from three electrodes in the experiment.

|  |  | Fz | | | | FCz | | | | Cz | | | |
| --- | --- | --- | --- | --- | --- | --- | --- | --- | --- | --- | --- | --- | --- |
| Subject No. | Group | Low load-congruent | Low load-incongruent | High load-congruent | High load-incongruent | Low load-congruent | Low load-incongruent | High load-congruent | High load-incongruent | Low load-congruent | Low load-incongruent | High load-congruent | High load-incongruent |
| 1 | High-trait-anxious | -2.171 | -3.982 | 0.044 | -2.025 | -3.185 | -5.04 | -0.638 | -4.581 | -2.824 | -4.787 | -0.374 | -4.819 |
| 2 | High-trait-anxious | -2.138 | -3.546 | -0.802 | -4.497 | -2.53 | -5.02 | -2.038 | -5.614 | -2.085 | -3.667 | -2.447 | -4.897 |
| 3 | High-trait-anxious | -1.921 | -0.499 | 1.689 | -2.716 | -2.123 | -1.88 | 0.599 | -4.091 | -0.201 | -1.918 | 1.359 | -1.755 |
| 4 | High-trait-anxious | -3.714 | -5.211 | -2.422 | -6.51 | -3.955 | -4.52 | -2.306 | -5.296 | -3.321 | -3.792 | -1.414 | -3.316 |
| 5 | High-trait-anxious | 0.8 | -0.216 | -0.07 | -3.68 | 2.387 | 0.5 | 1.856 | -3.014 | 2.843 | 1.604 | 2.288 | 0.471 |
| 6 | High-trait-anxious | -4.449 | -5.469 | -2.438 | -6.763 | -0.506 | -1.4 | 0.226 | -3.634 | 2.374 | 1.411 | 8.415 | 1.43 |
| 7 | High-trait-anxious | -9.54 | -11.562 | -9.237 | -13.557 | -9.235 | -11.13 | -8.971 | -12.666 | -6.199 | -7.595 | -6.367 | -9.601 |
| 8 | High-trait-anxious | -1.909 | -2.731 | -0.557 | -4.117 | -2.809 | -2.4 | -1.441 | -4.633 | -1.577 | -0.357 | -0.678 | -2.655 |
| 9 | High-trait-anxious | -0.97 | -2.297 | -0.42 | -4.819 | -0.246 | -1.23 | 0.824 | -3.392 | 2.197 | 1.453 | 2.847 | -0.056 |
| 10 | High-trait-anxious | -4.776 | -6.723 | -3.682 | -6.878 | -5.168 | -7.21 | -7.152 | -10.149 | -4.811 | -8.433 | -8.696 | -9.875 |
| 11 | High-trait-anxious | -4.88 | -5.344 | -5.215 | -10.499 | -0.647 | -2.27 | -1.915 | -7.738 | 1.372 | 1.869 | -0.354 | 0.43 |
| 12 | High-trait-anxious | 2.845 | 3.962 | 4.697 | -1.009 | 1.029 | -1.23 | 0.641 | -4.686 | 2.704 | -0.525 | -0.008 | -1.966 |
| 13 | High-trait-anxious | -1.363 | -2.817 | -0.415 | -3.014 | -0.765 | -2.51 | -1.572 | -4.289 | 0.725 | -0.853 | 0.052 | -3.102 |
| 14 | High-trait-anxious | 0.347 | -4.284 | -0.133 | -6.011 | 0.978 | -3.03 | 0.462 | -4.946 | 2.613 | -0.445 | 2.719 | -0.772 |
| 15 | High-trait-anxious | 0.202 | -2.322 | -3.045 | -6.294 | -0.093 | -1.65 | -2.699 | -6.217 | 2.183 | 1.172 | -0.019 | -3.364 |
| 16 | High-trait-anxious | -3.313 | -3.49 | -1.748 | -6.078 | -4.168 | -5.76 | -2.849 | -7.496 | -3.52 | -5.7 | -2.624 | -4.222 |
| 17 | High-trait-anxious | -8.09 | -7.951 | -7.187 | -10.028 | -7.297 | -7.34 | -7.341 | -9.837 | -6.539 | -6.999 | -8.064 | -9.734 |
| 18 | High-trait-anxious | 0.882 | -1.864 | 1.326 | -2.274 | 0.36 | -1.52 | 0.94 | -2.408 | 0.089 | -1.25 | 0.593 | -1.235 |
| 19 | High-trait-anxious | -5.444 | -6.935 | -4.56 | -8.272 | -4.145 | -5.2 | -2.874 | -7.204 | -1.679 | -2.34 | 0.235 | -0.887 |
| 1 | Low-trait-anxious | 3.127 | 1.081 | 6.778 | 4.605 | 4.213 | 1.77 | 5.992 | 4.578 | 5.739 | 2.786 | 6.378 | 4.762 |
| 2 | Low-trait-anxious | -0.049 | -1.218 | -0.313 | -1.602 | -1.506 | -2.34 | -1.562 | -2.599 | -2.291 | -2.416 | -1.314 | -2.396 |
| 3 | Low-trait-anxious | 4.406 | 1.258 | -0.737 | -3.944 | 1.785 | -1.03 | -2.769 | -6.076 | -0.261 | -3.14 | -4.227 | -6.54 |
| 4 | Low-trait-anxious | -6.3 | -8.099 | -5.585 | -6.563 | -10.031 | -11 | -9.175 | -10.746 | -6.571 | -7.36 | -8.032 | -7.556 |
| 5 | Low-trait-anxious | -1.03 | -2.954 | -0.772 | -4.118 | 1.757 | 0.36 | 1.143 | -1.154 | 6.221 | 5.553 | 5.355 | 4.219 |
| 6 | Low-trait-anxious | -5.292 | -4.973 | -3.077 | -4.459 | -1.619 | -2.38 | -0.094 | -1.532 | 3.411 | 3.054 | 6.246 | 3.263 |
| 7 | Low-trait-anxious | -1.416 | -2.196 | -2.375 | -3.717 | -0.189 | -1.41 | -1.916 | -2.782 | 2.292 | 0.828 | -0.66 | -0.544 |
| 8 | Low-trait-anxious | -2.179 | -3.668 | -3.889 | -6.173 | 1.662 | 1.01 | -1.29 | -2.724 | 6.193 | 5.911 | 2.212 | 1.775 |
| 9 | Low-trait-anxious | -3.316 | -3.253 | -2.056 | -3.275 | -1.916 | -2.54 | -0.683 | -1.935 | 1.583 | 0.536 | 2.665 | 1.241 |
| 10 | Low-trait-anxious | -4.097 | -6.39 | -4.224 | -8.039 | -5.436 | -6.91 | -6.396 | -8.158 | -4.891 | -5.92 | -6.339 | -6.376 |
| 11 | Low-trait-anxious | -5.204 | -5.47 | -5.306 | -7.612 | -4.657 | -5.51 | -4.982 | -7.087 | -2.623 | -3.421 | -3.359 | -5.56 |
| 12 | Low-trait-anxious | -4.926 | -6.149 | -5.843 | -8.012 | -4.647 | -6.27 | -5.08 | -7.402 | -2.348 | -3.469 | -2.672 | -4.866 |
| 13 | Low-trait-anxious | -2.393 | -2.729 | -2.172 | -3.63 | -2.012 | -3.17 | -2.034 | -3.436 | -1.426 | -2.195 | -1.556 | -2.436 |
| 14 | Low-trait-anxious | -2.154 | -2.891 | -2.315 | -2.508 | -2.132 | -3.01 | -2.757 | -2.618 | -1.04 | -1.9 | -1.576 | -1.508 |
| 15 | Low-trait-anxious | -0.237 | -2.406 | 0.426 | -0.608 | -0.803 | -2.08 | 0.045 | -2.367 | 0.479 | 0.073 | 0.344 | -1.554 |
| 16 | Low-trait-anxious | -7.283 | -7.69 | -9.081 | -9.449 | -6.472 | -7.3 | -8.004 | -8.448 | -5.002 | -5.815 | -6.418 | -6.268 |
| 17 | Low-trait-anxious | -1.773 | -2.245 | -4.405 | -5.419 | -0.773 | -2.01 | -3.852 | -4.902 | 0.242 | 5.384 | -2.955 | -4.23 |
| 18 | Low-trait-anxious | 0.956 | -0.888 | 1.419 | 0.17 | -0.642 | -1.68 | 0.024 | -0.73 | 1.245 | 0.209 | -0.204 | -0.794 |
