# Supplementary material for: Impact of Working Memory Load on Cognitive Control in Trait Anxiety: An ERP Study
Source: PLoS One. 2014 Nov 4;9(11):e111791. doi: 10.1371/journal.pone.0111791 (PMC4219777; doi:10.1371/journal.pone.0111791)
Supplement: Table S5 — N2 latencies (ms) data recorded from three electrodes in the experiment. (DOC) [file pone.0111791.s005.doc]

Table S5. N2 latencies (ms) data recorded from three electrodes in the experiment.

|  |  | Fz | | | | FCz | | | | Cz | | | |
| --- | --- | --- | --- | --- | --- | --- | --- | --- | --- | --- | --- | --- | --- |
| Subject No. | Group | Low load-congruent | Low load-incongruent | High load-congruent | High load-incongruent | Low load-congruent | Low load-incongruent | High load-congruent | High load-incongruent | Low load-congruent | Low load-incongruent | High load-congruent | High load-incongruent |
| 1 | High-trait-anxious | 284 | 264 | 290 | 306 | 274 | 266 | 270 | 268 | 270 | 266 | 262 | 268 |
| 2 | High-trait-anxious | 328 | 346 | 288 | 328 | 324 | 348 | 284 | 324 | 304 | 350 | 280 | 288 |
| 3 | High-trait-anxious | 320 | 326 | 322 | 330 | 322 | 330 | 322 | 332 | 322 | 332 | 318 | 254 |
| 4 | High-trait-anxious | 292 | 358 | 292 | 316 | 292 | 298 | 290 | 298 | 290 | 294 | 288 | 296 |
| 5 | High-trait-anxious | 378 | 374 | 334 | 326 | 328 | 328 | 338 | 328 | 378 | 328 | 340 | 330 |
| 6 | High-trait-anxious | 270 | 272 | 258 | 270 | 268 | 268 | 252 | 268 | 250 | 268 | 350 | 250 |
| 7 | High-trait-anxious | 346 | 360 | 350 | 344 | 348 | 358 | 350 | 364 | 348 | 356 | 350 | 368 |
| 8 | High-trait-anxious | 276 | 306 | 334 | 290 | 274 | 278 | 284 | 290 | 268 | 268 | 266 | 286 |
| 9 | High-trait-anxious | 300 | 312 | 310 | 314 | 300 | 304 | 306 | 310 | 300 | 300 | 304 | 308 |
| 10 | High-trait-anxious | 350 | 364 | 350 | 350 | 350 | 366 | 350 | 362 | 350 | 366 | 350 | 366 |
| 11 | High-trait-anxious | 314 | 316 | 316 | 320 | 306 | 312 | 264 | 314 | 250 | 308 | 250 | 310 |
| 12 | High-trait-anxious | 370 | 304 | 362 | 370 | 356 | 370 | 368 | 368 | 280 | 366 | 364 | 360 |
| 13 | High-trait-anxious | 284 | 286 | 332 | 268 | 258 | 272 | 264 | 270 | 250 | 268 | 258 | 270 |
| 14 | High-trait-anxious | 388 | 378 | 370 | 380 | 390 | 378 | 370 | 378 | 390 | 378 | 374 | 378 |
| 15 | High-trait-anxious | 358 | 330 | 342 | 354 | 360 | 334 | 344 | 352 | 348 | 332 | 342 | 348 |
| 16 | High-trait-anxious | 318 | 316 | 294 | 300 | 320 | 318 | 296 | 302 | 268 | 312 | 292 | 304 |
| 17 | High-trait-anxious | 280 | 326 | 262 | 318 | 276 | 304 | 260 | 316 | 270 | 322 | 258 | 318 |
| 18 | High-trait-anxious | 248 | 252 | 254 | 254 | 242 | 244 | 248 | 248 | 238 | 238 | 244 | 240 |
| 19 | High-trait-anxious | 282 | 286 | 326 | 338 | 274 | 284 | 326 | 308 | 250 | 250 | 326 | 338 |
| 1 | Low-trait-anxious | 296 | 326 | 294 | 316 | 296 | 328 | 304 | 320 | 296 | 330 | 310 | 322 |
| 2 | Low-trait-anxious | 276 | 300 | 278 | 282 | 272 | 302 | 274 | 284 | 268 | 294 | 268 | 278 |
| 3 | Low-trait-anxious | 300 | 318 | 300 | 300 | 300 | 306 | 300 | 300 | 300 | 306 | 300 | 300 |
| 4 | Low-trait-anxious | 330 | 318 | 304 | 336 | 258 | 330 | 276 | 338 | 332 | 336 | 264 | 336 |
| 5 | Low-trait-anxious | 338 | 352 | 342 | 354 | 336 | 352 | 342 | 354 | 332 | 346 | 338 | 352 |
| 6 | Low-trait-anxious | 302 | 322 | 306 | 322 | 300 | 324 | 306 | 326 | 298 | 326 | 300 | 330 |
| 7 | Low-trait-anxious | 336 | 356 | 336 | 344 | 330 | 342 | 334 | 342 | 326 | 338 | 332 | 342 |
| 8 | Low-trait-anxious | 326 | 338 | 326 | 332 | 324 | 338 | 320 | 328 | 320 | 332 | 308 | 320 |
| 9 | Low-trait-anxious | 332 | 332 | 326 | 322 | 330 | 330 | 326 | 322 | 330 | 330 | 326 | 320 |
| 10 | Low-trait-anxious | 330 | 346 | 354 | 346 | 332 | 346 | 360 | 346 | 272 | 266 | 354 | 346 |
| 11 | Low-trait-anxious | 356 | 354 | 360 | 342 | 356 | 354 | 344 | 342 | 352 | 354 | 344 | 342 |
| 12 | Low-trait-anxious | 284 | 280 | 286 | 290 | 278 | 282 | 286 | 294 | 250 | 284 | 258 | 296 |
| 13 | Low-trait-anxious | 310 | 298 | 308 | 308 | 308 | 300 | 308 | 306 | 304 | 298 | 306 | 298 |
| 14 | Low-trait-anxious | 282 | 276 | 274 | 284 | 282 | 274 | 276 | 282 | 278 | 266 | 276 | 278 |
| 15 | Low-trait-anxious | 358 | 366 | 362 | 362 | 350 | 360 | 364 | 360 | 350 | 350 | 366 | 358 |
| 16 | Low-trait-anxious | 280 | 278 | 278 | 288 | 276 | 272 | 274 | 286 | 270 | 268 | 270 | 282 |
| 17 | Low-trait-anxious | 224 | 210 | 236 | 226 | 222 | 208 | 226 | 224 | 208 | 312 | 204 | 212 |
| 18 | Low-trait-anxious | 310 | 314 | 312 | 308 | 310 | 312 | 310 | 306 | 308 | 310 | 308 | 304 |
